# Multilayered hyperbolic Au/TiO_2_ nanostructures for enhancing the nonlinear response around the epsilon-near-zero point

**DOI:** 10.3762/bjnano.17.17

**Published:** 2026-02-05

**Authors:** Fernando Arturo Araiza-Sixtos, Mauricio Gomez-Robles, Rafael Salas-Montiel, Raúl Rangel-Rojo

**Affiliations:** 1 Department of Optics, Applied Physics Division, Centro de Investigación Científica y de Educación Superior de Ensenada, B.C., México; 2 Laboratoire Lumière, nanomatériaux, nanotechnologies - L2n CNRS UMR 7076, Université de Technologie de Troyes, 10004 Troyes, France; 3 EUT+ Institute of Nanomaterials and Nanotechnologies-EUTINN, European University of Technology, European Union

**Keywords:** epsilon-near-zero (ENZ), hyperbolic metamaterials, nonlinear optics (NLO)

## Abstract

In this work, we present the design, fabrication, and study of the optical properties of multilayered metal–dielectric Au/TiO_2_ structures. The samples were fabricated using Joule effect evaporation for gold and electron beam evaporation for titanium dioxide. Their structure was designed to have an epsilon-near-zero (ENZ) point at different wavelengths around 800 nm, in order to study their nonlinear response as a function of the resonance conditions around the ENZ point. The characterization of the linear properties of the samples was done using spectrophotometry and spectral ellipsometry. We studied the nonlinear response with the z-scan technique at different incident irradiances using a Ti:sapphire femtosecond laser, enabling us to characterize both the refractive and absorptive contributions to the nonlinear response. Due to the high pulse repetition rate inherent to Ti:sapphire systems and the presence of linear absorption in the samples, cumulative pulse-to-pulse thermal effects may be present. A modified version of the z-scan technique that allowed us to separate the electronic from the thermal contribution was used. A clear enhancement of the nonlinear response was observed for the sample with an ENZ point around the laser wavelength 800 nm with a nonlinear refractive index of *n*_2_ = 0.103 ± 0.006 cm^2^·GW^−1^, a value that is comparable to other ENZ materials in literature.

## Introduction

In recent years, the invention of new techniques to fabricate nanostructured materials led to the creation of metamaterials. These new materials combine known materials in arrays whose geometries are easy to manipulate in order to have properties that are different from those of the constituting materials. The principal characteristic of metamaterials is that the internal structures of the constituents are much smaller than the wavelength of light. As a consequence, light does not differentiate between the constituting materials and only “sees” them as a single material. To study the interaction of light with these metamaterials, we use the effective media approximation to calculate the optical properties of these materials. These properties are completely different than those of the constituting materials and are not simply an average of them [1].

A type of metamaterials that we are particularly interested in are hyperbolic metamaterials (HMMs), whose dispersion relation generates an hyperboloid in the k-space. There are some readily available materials in nature that present hyperbolic dispersion, such as bismuth and 2D graphene sheets [2], indium tin oxide (ITO) [3], and aluminum-doped zinc oxide (AZO) [4]. HMMs are highly anisotropic and exhibit some interesting properties, including strong enhancement of spontaneous emission, negative refractive index, and near-zero permittivity. The main benefit of HMMs is that, with the correct manipulation of the design, we are able to choose the wavelength in which we would like these phenomena to appear. Hyperbolic dispersion can be achieved by combining a dielectric, which has a permittivity that is always positive, and a metal, which has a negative real part of the permittivity below the plasma frequency. The properties of the new material depend on the constituting materials and the geometry of the design.

The property of this type of materials we are interested in for this work is that, with the right manipulation of the geometry, we are able to make the electric permittivity near zero (“epsilon near zero”, ENZ). The ENZ point causes light to travel through the medium with constant phase, it enhances the electric field, and it enhances the nonlinear response by combining coherently the response generated at different planes within the medium without the need of phase matching [5]. Ideally, we would like these materials not to have linear absorption, which prevents the full exploitation of these properties. The presence of linear absorption means that ℑ{ε} ≠ 0, which precludes the magnitude of the permittivity to become completely zero, hence the term “epsilon near zero”. Also, we are able to make ℜ{ε} arbitrarily close to zero; as a consequence, we have a refractive index *n*_0_ that is near zero. This near-zero refractive index allows us to enhance the nonlinear refractive index because *n*_2_ ∝ 1/*n*_0_^2^.

Various structures exhibiting hyperbolic dispersion have been fabricated and analyzed. The first experimental realization of a layered hyperbolic material took the form of a hyperlens [6]. In 2012, a significant advancement was made by Subramania et al., who fabricated layered Ag/TiO_2_ and determined via spectroscopy that its ENZ point laid within the visible spectrum [7]. Since then, a wide array of layered metal–dielectric configurations have been demonstrated, including silver-based combinations such as Ag/SiO_2_ [1], Ag/Al_2_O_3_ [6], Ag/LiF [8], Ag/TiO_2_ [9], Ag/Ge [10], Ag/MgF_2_ [11], and Ag/Ti_3_O_5_ [12]. Gold-based systems have also been prominent, such as Au/Al_2_O_3_ [13–15], Au/SiO_2_ [16], Au/TiO_2_ [17], and optical filters based on Au/TiO_2_ multilayers [18]. Beyond classic metal–dielectric structures, research has explored ZnO/Al:ZnO [19] and doped semiconductors [20]. Work has also expanded into complex geometries, such as hybrid metal–dielectric layered pyramids [10], AZO nanotrenches for molecular absorption sensing [4], and the first nanowire array exhibiting hyperbolic properties with negative refraction at 780 nm [21].

Previously, our group has studied the nonlinear response of a Ru/TiO_2_ multilayered structure as reported in [22]. This sample was designed to have an ENZ point at 800 nm and was fabricated using atomic layer deposition. During the linear characterization of the optical properties, spectral ellipsometry showed that the ENZ point actually was not at 800 nm, but rather at 600 nm. We tried to find the reason of this shift with our available resources. Transmission electron microscopy showed that there was no significant change in the geometry, which could have resulted in the ENZ shift. We could not study the composition of the structure so we could only assume that, during the fabrication process, the deposited Ru was oxidized, and the permittivity of the material substantially changed. We studied the nonlinear response of the structure using a tunable femtosecond source and found that, even with the shift in the ENZ point seen with ellipsometry, there was a clear enhancement at 800 nm. Unfortunately, due to time constraints, we could not study the nonlinear response at higher wavelengths; thus, we could not corroborate if this was a local maximum or a value found in the tail of the local maximum at a higher wavelength.

Considering our previous findings, in this work, we decided to change the fabrication methods and the metal used to avoid possible contamination due to oxidation. We chose to use gold instead of ruthenium because it is easier to deposit and has the correct permittivity to create a HMM. Gold also has a low oxidation rate, allowing us to avoid any changes in permittivity due to oxidation during the fabrication process. We kept using titanium dioxide because, during the simulation stage, we found that its permittivity allowed us to keep the overall sample thickness low. Also, during the fabrication process, we found that we could avoid problems like lack of adherence between the layers. We also decided that, instead of relying on a tunable femtosecond source, we would manipulate the material geometry to design different samples with an ENZ point at different wavelength values around 800 nm, that is, we will tune the material to the laser wavelength rather than the other way around.

We make a characterization of the linear optical properties of the samples and present a study of the nonlinear optical properties. For the linear characterization of the samples, we study the absorption using spectrophotometry and the complex effective permittivity using spectroscopic ellipsometry. Using a Ti:sapphire femtosecond pulsed laser, we study the third-order nonlinear response of the samples employing the z-scan technique [23].

Even though the use of femtosecond pulses allows us to avoid any thermal load to the sample due to absorption, the high repetition rate intrinsic to the Ti:sapphire oscillators leads to the possible accumulation of pulse-to-pulse thermal load. This thermal effect needs to be quantified. In order to do so, we modified the z-scan technique, adding a variable frequency chopper to limit the number of pulses incident on the sample without modifying the incident peak irradiance. The thermal load to the sample is reduced with the increase of the chopper’s frequency while the electronic contribution to the third order nonlinear response is still excited. In this way, we can resolve the electronic and thermal contributions to the response as explained in [24]. We present results that enabled us to determine the thermal nonlinear response due to intrapulse heating.

## Design and Fabrication of Nanostructured Metal–Dielectric HMM

**Figure 1:**
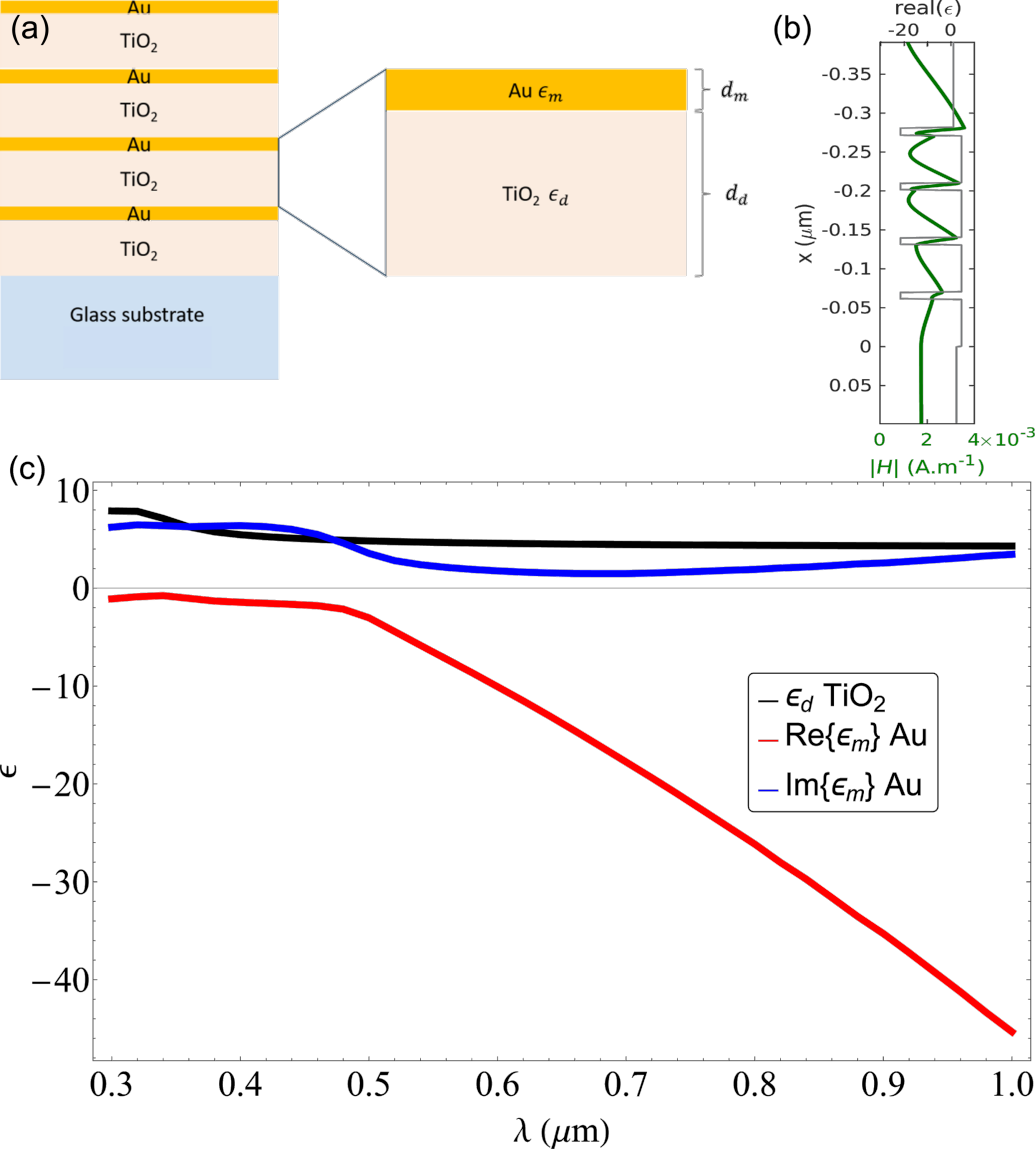
(a) Bilayer structure design. The metal and dielectric layers are characterized, respectively, by their permittivity ε_m_ and ε_d_ and their widths *d*_m_ and *d*_d_. (b) Spatial distribution of the electric field inside the multilayer. (c) Permittivity of gold and titanium dioxide reported in literature [26–27].

For a multilayer system with layer widths of 10–100 nm, we can employ the effective medium approximation (EMA) to calculate the properties of the whole system. It is important to note that the layers must follow a metal–dielectric repetition sequence. For a metal and a dielectric with permittivities ε_m_ and ε_d_, respectively, it is possible to obtain a relation for the principal components 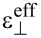 and 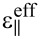 of the composite, which are given by [25]:


[1]

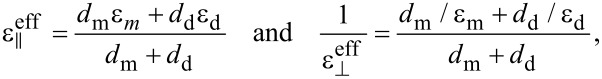



where *d*_m_ and *d*_d_ are the widths of the metallic and dielectric layers, respectively, as seen in Figure 1a.

The z-scan technique employed to study the nonlinear properties of the samples involves normal incident light, where the only relevant component is 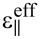; hence, we only calculate this component. We wish to produce samples with four metal–dielectric periods that have different ENZ wavelength values (λ = 740, 760, 780, 800, 820, 840, and 860 nm) to test the effect of the resonance conditions with the laser wavelength on the nonlinear effects. We also produced a set of single layers to study the properties of the constituting materials being deposited.

In order to calculate the point for which 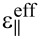 = 0, we use the permittivities of gold ε_m_ and titanium dioxide ε_d_ from [26–27], shown in Figure 1c. This leaves the layer thicknesses *d*_m_ and *d*_d_ as the only parameters to design the structures. In order to minimize losses due to absorption, we chose a fixed width as small as possible of the metallic layer *d*_m_; then, we solve for 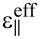 = 0 to obtain the width of the dielectric layer *d*_d_, which will give the ENZ at the desired wavelength. It has been found that layers of gold with a thickness below 10 nm would lead to an inhomogeneous layer with separated gold “islands” throughout the surface rather than a continuous layer [28]. Because of this, we chose to use a gold layer thickness of 10 nm, the limit to have uniform layers found in literature. We choose four metal–dielectric periods to also reduce absorption due to the gold and because it has been shown that using more than five periods produces no appreciable improvement in the nonlinear optical response [29].

**Figure 2:**
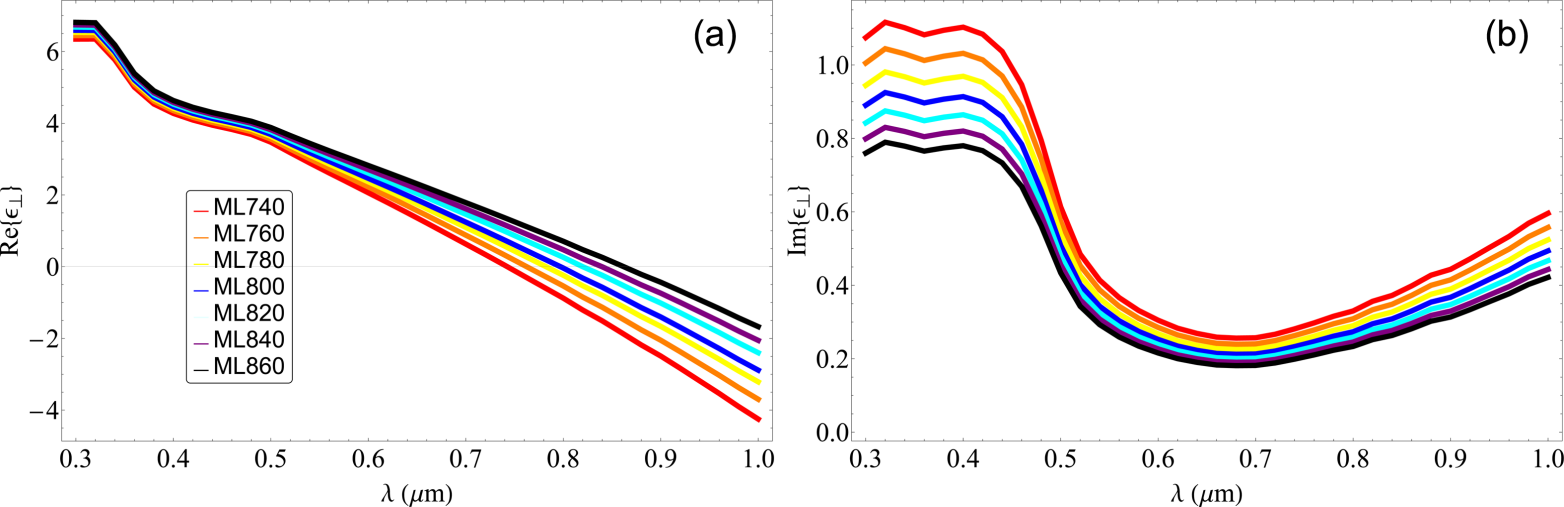
(a) Simulated real part of the perpendicular component of the permittivity of the Au/TiO_2_ multilayered material having a fixed width of the metallic layer *d*_m_ = 10 nm with varying thickness of the dielectric layer. (b) Simulated imaginary part of the perpendicular component of the permittivity of the Au/TiO_2_ multilayered material.

In Table 1, we can see the thickness for the dielectric layers needed to yield an ENZ point at the desired wavelengths, calculated solving for 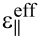 = 0. We then simulate the permittivity of the stack as a whole. In Figure 2a, we present the real part of the perpendicular component of the effective permittivity; we can see the shift in the ENZ point for every multilayer due to the varying thickness of the dielectric layer. In Figure 2b, we present the imaginary part of the perpendicular component and see that ℑ{ε}, which is related to absorption, has a small value around 800 nm; hence, we can expect to have minimum losses in the designed materials.

**Table 1:** Thicknesses of metallic and dielectric layers and their correspondent ENZ wavelengths.

Sample	Nominal			Experimental		

	λ_ENZ_ (nm)	*d*_m_ (nm)	*d*_d_ (nm)	λ_ENZ_ (nm)	*d*_m_ (nm)	*d*_d_ (nm)

ML740	740	10	48	730	13	52
ML760	760	10	52	789	11	57
ML780	780	10	56	780	12	58
ML800	800	10	60	803	12	62
ML820	820	10	64	842	12	69
ML840	840	10	68	862	11	69
ML860	860	10	72	892	11	77

## Methods

The third-order nonlinear optical properties of the material were explored first using the z-scan technique [23] with femtosecond pulses at 800 nm from a Ti:sapphire oscillator. The laser employed in our studies is a Coherent Mira-900 mode-locked Ti:sapphire oscillator, which produces 100 fs pulses with a 76 MHz repetition rate. It has a spectral width of Δλ = 10 nm centered around 800 nm with very little to no tuning capability. For the z-scan technique, a 200 mm focal length lens was used to focus the beam on the sample. The waist of the beam at the focal plane was ω_0_ = 28 μm. Using an aperture before the detector, we are able to study both the nonlinear absorption when the aperture is fully open (open aperture, OA), and the nonlinear refraction when the aperture is partially open (closed aperture, CA).

To study the possible thermal contribution to the nonlinear response due to pulse-to-pulse temperature accumulation, we used a modified version of the z-scan technique, previously reported in [30]. This modification consists in the addition of a chopper with a duty cycle of 1:8, which will limit the incident pulses to the sample as a function of its frequency. The addition of this chopper will allow us to reduce the thermal load to the sample due to intrapulse heating whilst keeping the peak irradiance constant to still be able to excite the electronic contribution of the nonlinear response. In this way, the thermal effect will change with the chopper frequency, while the electronic contribution, function only of the input irradiance, will remain constant.

We fabricated the ENZ materials at the nano’mat platform, University of Technology at Troyes, France. Using a Plassys MEB400 evaporator, we deposited TiO_2_ and Au layers on glass substrates without breaking vacuum. TiO_2_ layers were evaporated via electron beam and Au layers via Joule effect deposition, both at a deposition rate of 0.15 nm·s^−1^ and a base pressure of 2 µTorr. The target thickness for Au was 10 nm, while that of TiO_2_ was varied from 44 to 72 nm to achieve ENZ properties at or around the working wavelength of 800 nm. Every structure consisted of four metal–dielectric periods, leaving us with a total of eight layers.

We verified the total thickness of the multilayer stack using a Bruker Dektak XT profilometer. We used a Hach DR6000 UV–vis spectrophotometer to measure the linear absorption of each sample over the range from 300 to 1000 nm. Optical constants of Au and TiO_2_ layers were measured via variable-angle spectroscopic ellipsometry (J.A. Woollam M-2000) over a wavelength range from 350 to 1650 nm and over an angular range from 45° to 70° in 5° steps. Even though we studied the effective permittivity in a wider range, we are only interested in the visible region because it is the region in which we are able to study the nonlinear response. Data analysis and multilayer modeling were performed using CompleteEASE software by J.A. Woollam. The optical constants were fitted using a B-splines layer with a Au thin film with KK oscillator as a starting material for Au, and with TiO_2_ with Cody–Lorentz oscillators as starting material for TiO_2_.

Scanning electron microscopy (Hitachi SU8030) was used to obtain the cross-sectional image (below in Figure 4a), with the sample oriented to expose the ENZ material edge to the electron beam.

## Results and Discussion

### Linear characterization

**Figure 3:**
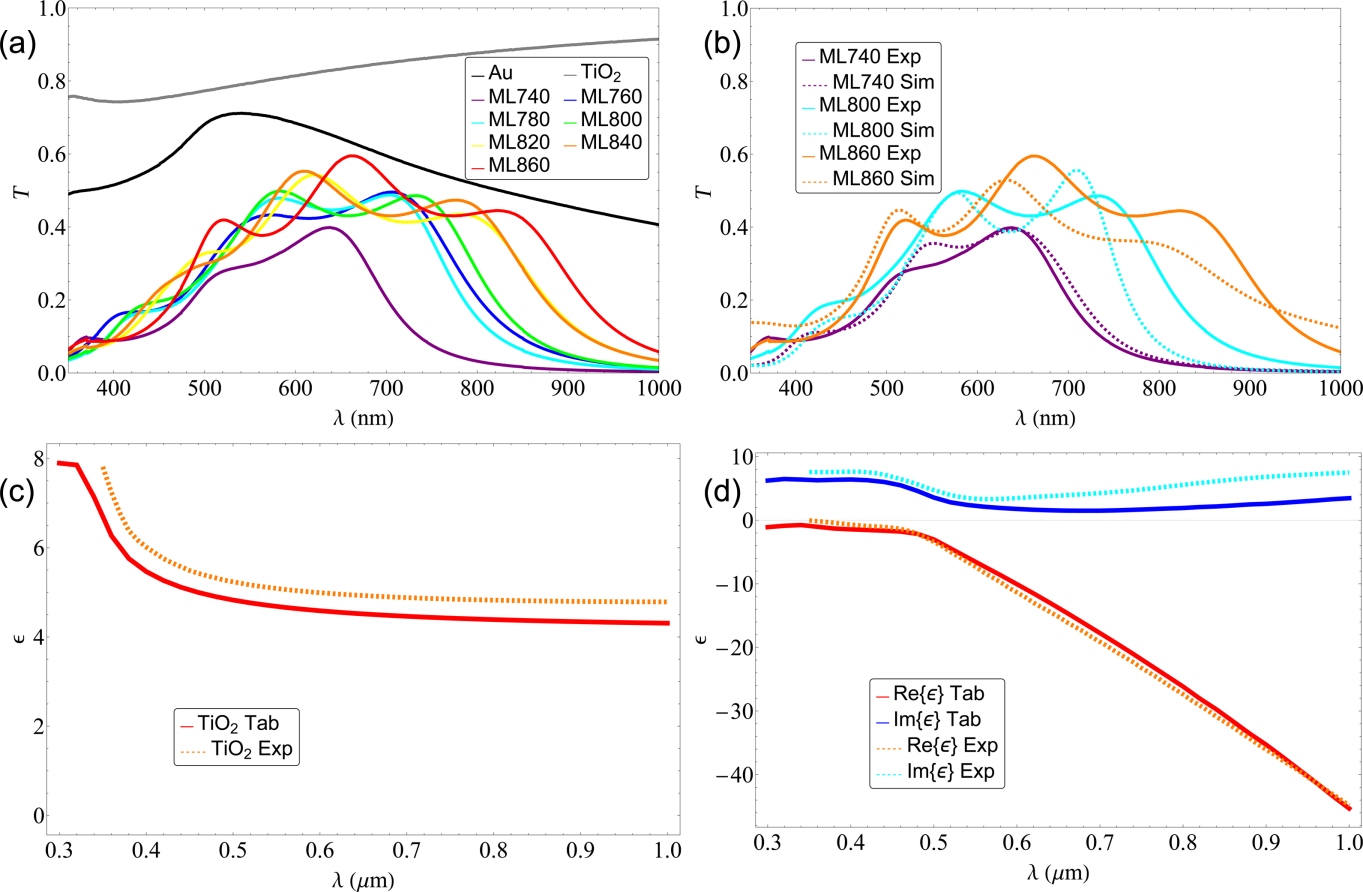
(a) Transmittance of the stacks with fixed gold layer *d*_m_ = 10 nm measured using spectrophotometry. We can observe a relatively high absorbance around 800 nm with transmittances down to 40%. (b) Comparison of the measured spectrum against the simulated spectrum for the samples ML740 (purple), ML800 (cyan), and ML860 (orange). (c) Permittivity of a single *d*_d_ = 60 nm titanium dioxide layer measured using spectral ellipsometry compared to the one reported in literature. We observe that the resulting permittivity is almost similar to the one used for the simulations. (d) Permittivity of a single *d*_m_ = 10 nm gold layer measured using spectral ellipsometry compared to the one reported in literature. We observe that the resulting permittivity is almost similar to the one used for the simulations.

The linear absorption of the sample was studied using spectrophotometry to measure the transmittance of our samples (Figure 3a). We see that the structures have an absorption edge present in the UV region, which probably has a strong contribution from the glass substrate, then a dip in the visible region, and finally an increase towards the IR region. We can observe a relatively high absorbance around 800 nm with transmittances down to 40%, which could lead to losses and undesired thermal effects.

**Figure 4:**
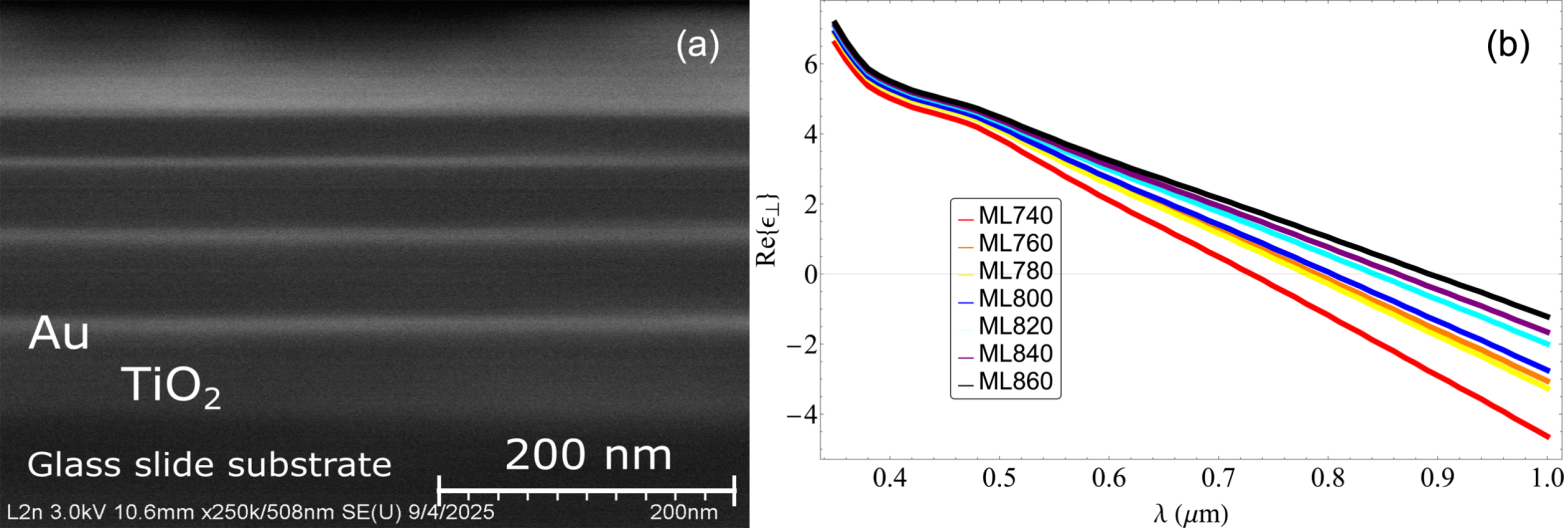
(a) Scanning electron microscopy image of the ML800 structure. We see that the deposited layers have different thicknesses throughout the structure, resulting in a shift in the ENZ points. (b) Calculated permittivity using the results from the ellipsometry. We observe that, although the ENZ points shifted, they still remain near the region of 800 nm.

The complex permittivity of single layers of the constituting materials was studied by variable angular spectroscopic ellipsometry. Figure 3c shows the measured permittivity of the titanium dioxide compared to the values from literature. We observe that the values are very similar, indicating that the deposited film is almost the same as the one used for the initial simulations. Figure 3d shows the measured permittivity of the thin gold layer compared to the values of ℜ{ε} and ℑ{ε} from literature; they are very similar, indicating that the gold layers have a good morphology with no voids. Also using ellipsometry, we were able to measure the thicknesses of the deposited layers. In Table 1, we can see that we have widths for every stack that are different from the ones proposed for the simulated ENZ points. This change in thickness was also seen in scanning electron microscopy (SEM). In Figure 4a we present a SEM image of the ML800 structure; we see that the layers deposited have different thicknesses throughout the structure. Adding the change in permittivity of the deposited gold and titanium dioxide layers, together with the changes layer thickness, we repeated the simulations to see where the ENZ points will be.

With the measured permittivities and layer thicknesses, we simulated the transmittance of the multilayer structures using EMUstack. Figure 3b shows a comparison of the measured spectrum with the simulated spectrum for the samples ML740 (purple), ML800 (cyan), and ML860 (orange). We observe a good agreement with the experimental data with some differences in the positions and values of the different maxima observed. This similitude tells us that no contamination occurred during the deposition process and that the deposited materials have almost identical properties.

Also, with the measured thicknesses and permittivities of the deposited layers, we repeated the simulations in order to check if the ENZ points remain the same. In Figure 4b, we present the new simulated permittivities, and in Table 1, we can observe that the ENZ wavelengths have changed for every stack. Although the ENZ points have shifted slightly, they still remain near the region of 800 nm. Hence, we expect to be able to study the enhancement of the nonlinear response as a function of the ENZ position.

### Nonlinear optical studies

We started studying the nonlinear response using the z-scan technique at different input peak irradiances, namely, 0.54, 0.60, 0.68, 0.82, 0.96, and 1.09 GW·cm^−2^. We studied the nonlinear response as a function of the input peak irradiance in order to reduce the error in the determination of the nonlinear parameters when only using a single measurement.

We performed the z-scan for the single layers of the constituting materials. For both gold and titanium dioxide, we could not see a signal. This could mean that our system lacks the power output to excite the nonlinear response or that, even at our highest irradiance, the response was below the signal-to-noise ratio. Considering that, for 800 nm, our system has a minimum resolution of 2%, we can calculate an upper limit for the nonlinear refractive index that corresponds to a peak-to-valley transmittance difference of 0.02 (Δ*T*_P−V_ = 0.02) giving us 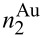 ≤ 0.024 cm^2^·GW^−1^ for gold and 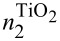 ≤ 4.4 × 10^−6^ cm^2^·GW^−1^ for titanium dioxide.

**Figure 5:**
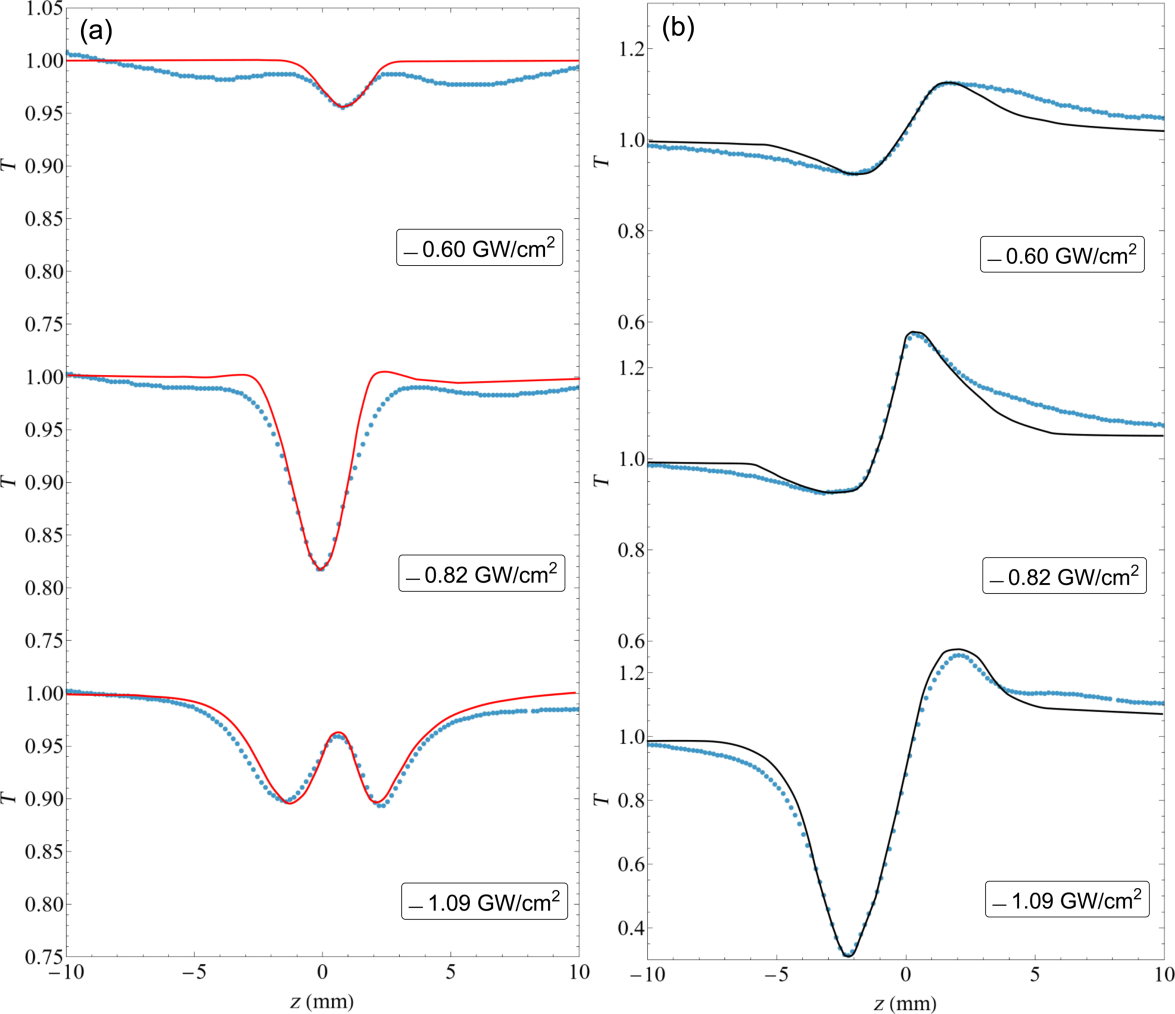
(a) OA z-scan results. The solid line is the fit using the method presented in [31]; we successfully emulated the signal with two effects of different sign. (b) For the CA z-scan results, we could produce a fit that is very similar to the experimental data.

First, we performed the OA z-scan measurements. Figure 5a shows the results obtained for three different input irradiances in the multilayer stack with an ENZ point at 800 nm. Usually, we should observe a signal that grows for high *I* at *z* = 0 in the case of saturable absorption or that diminishes in the case of induced absorption. What we observe in Figure 5a is a combination of two processes, namely, induced and saturable absorption, which change their relative contributions for the different input irradiances. Then we performed the CA z-scan. The results are presented in Figure 5b and show the presence of a positive *n*_2_, that is, a prefocal minimum followed by a postfocal maximum, albeit somehow deformed. This deformation is due to the complicated nonlinear absorption observed in the OA z-scan.

In order to model the response, we used the approach found in [31], where the CA z-scan trace shows effects of both nonlinear absorption and refraction:


[2] $$T=1+\frac{{\left(1-S\right)}^{\mu }\sin(\xi )}{S\left(1+z^{2}\right)}\Delta \Phi_{0}-\frac{1-{\left(1-S\right)}^{\mu }\cos\left(\xi \right)}{S\left(1+z^{2}\right)}\Delta \Psi_{0},$$

[3] $$\mu =\frac{2\left(z^{2}+3\right)}{z^{2}+9},$$

[4] $$\xi =\frac{-4z\ln\left(1-S\right)}{z^{2}+9},$$

[5] $$\Delta \Phi_{0}=\frac{2\pi }{\lambda }n_{2}I_{0}L_{\text{e}},$$

[6] $$\Delta \Psi_{0}=\frac{\beta I_{0}L_{\text{e}}}{2\sqrt{2}},$$

where *S* is the aperture transmittance, *I*_0_ is the peak irradiance, and 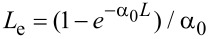 is the effective length. Setting *S* = 1, we are left with ΔΨ_0_, which is related only to nonlinear absorption effects, and we can then model the fit for the OA z-scan results. For the CA z-scan, we set *S* = 0.5 (from experiment), and we only need to find the value for ΔΦ_0_ to fit the experimental data using the ΔΨ_0_ values from the previous OA z-scan fits.

It can be noted that Equation 6 describes a single nonlinear absorption effect. However, since we observed the combination of two effects of opposite sign, we must modify β to describe both effects. Substituting


[7] $$\beta \left(I\right)=\beta_{\text{TPA}}\left(I\right)+\beta_{\text{SA}}\left(I\right)=\frac{\beta_{+}}{1+\frac{I}{I_{S}^{+}}}+\frac{\beta_{-}}{1+\frac{I}{I_{S}^{-}}}$$

in Equation 6, we are now able to model the effect of both nonlinear absorption effects.

Then, for each different sample, we must find a set of constants β_+_, β_−_, 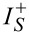, and 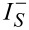 that can be used to fit every z-scan data set obtained at different *I*_0_ values. Figure 5 shows the fits made to the previous data set using Equation 2, showing good agreement with the experimental data. For the CA z-scan, we used the nonlinear absorption fit parameters from the OA z-scan data. We then fit ΔΦ_0_ to model the CA z-scan; the results are shown in Figure 5, and the corresponding *n*_2_ parameters are given in Table 2.

**Table 2:** Nonlinear properties (fit parameters for CA z-scan) of the multilayered stacks with *d*_m_ = 10 nm.

Sample	*n*_2_ (×10^−2^ cm^2^·GW^−1^)	β_+_ (×10^4^ cm·GW^−1^)	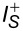 (GW·cm^−2^)	β_−_ (×10^3^ cm·GW^−1^)	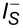 (GW·cm^−2^)

ML740	0.53 ± 0.03	4.9 ± 0.3	1.37 ± 0.12	−1.32 ± 0.09	0.026 ± 0.005
ML760	0.21 ± 0.06	5.0 ± 0.5	1.26 ± 0.05	−1.47 ± 0.07	0.038 ± 0.004
ML780	3.04 ± 0.03	4.7 ± 0.1	1.13 ± 0.08	−1.26 ± 0.02	0.031 ± 0.011
ML800	10.3 ± 0.4	5.2 ± 0.2	0.980 ± 0.015	−0.88 ± 0.01	0.035 ± 0.005
ML820	6.9 ± 0.09	4.2 ± 0.4	0.986 ± 0.007	−0.72 ± 0.07	0.039 ± 0.005
ML840	0.51 ± 0.04	4.7 ± 0.2	0.973 ± 0.012	−0.99 ± 0.05	0.024 ± 0.003
ML860	0.064 ± 0.006	4.3 ± 0.1	0.994 ± 0.005	−0.82 ± 0.03	0.027 ± 0.009

**Figure 6:**
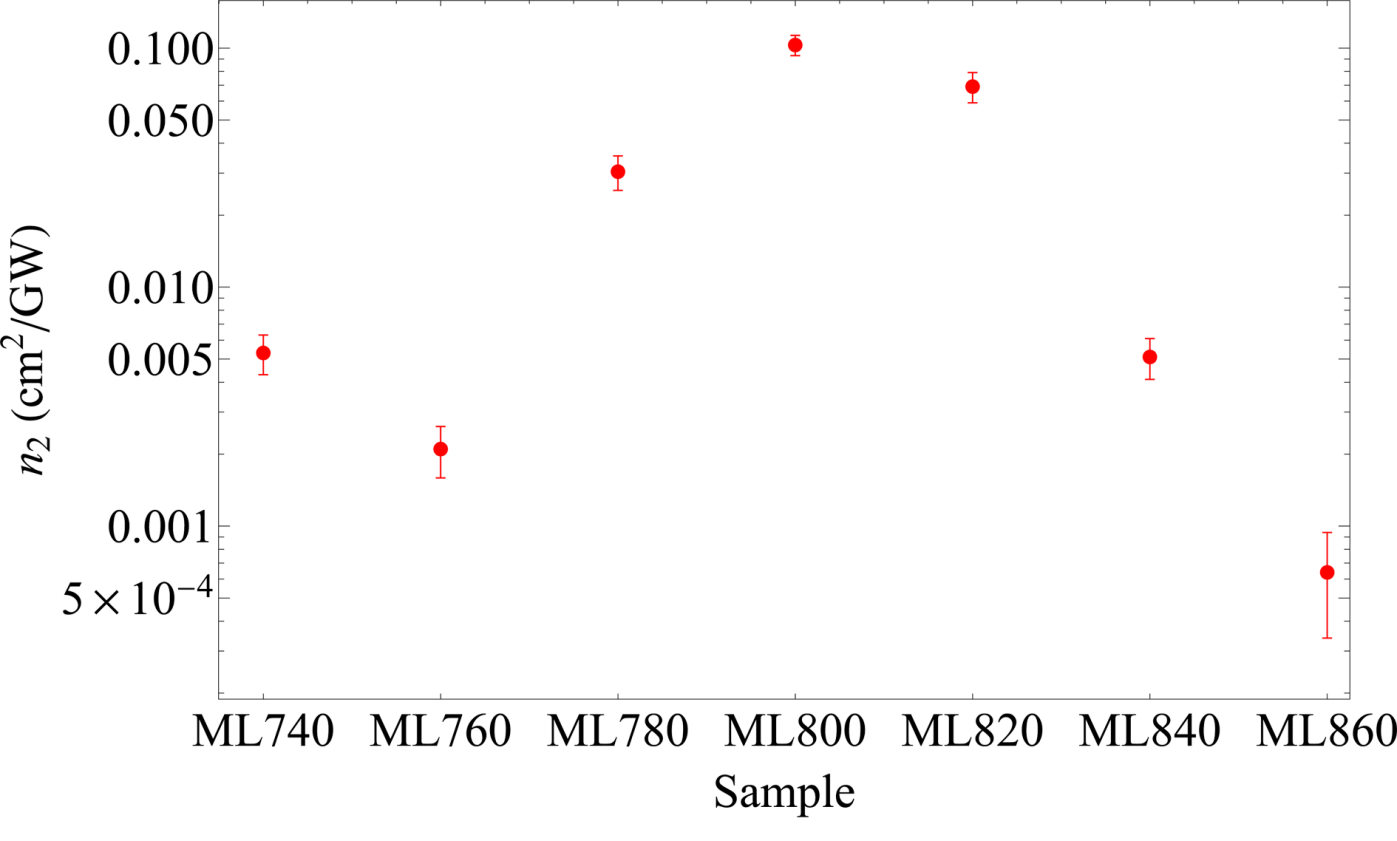
The nonlinear refractive index *n*_2_ as a function of the ENZ point wavelength. We found a clear enhancement around 800 nm, as desired.

We see that the *n*_2_ determined for the sample with the ENZ point at 800 nm is larger than all the others, by up to two orders of magnitude with respect to the ones with the smallest coefficients (Figure 6). This clearly indicates that the observed nonlinear response is enhanced by being resonant with the ENZ wavelength; compared to that of the constituting materials, it is five times larger than the measured *n*_2_ of gold and 10^4^ times larger than that of TiO_2_.

### Electronic and thermal contributions to the nonlinear response

We use ultrashort pulses due to the low pulse energies needed to achieve irradiances high enough to observe nonlinear effects. This avoids the presence of intrapulse thermal effects; therefore, only the electronic contribution is in principle measured. It has been shown in [24] that the presence of linear and nonlinear absorption and the high pulse repetition rate intrinsic to the Ti:sapphire oscillators (around 100 MHz) may produce pulse-to-pulse temperature accumulation. This new effect will produce a contribution to the nonlinear refractive index of thermal origin. Given its thermal origin, this contribution will have a relatively long characteristic time. Almost every application exploits the electronic contribution of the nonlinear response; hence, we must study and define the nature of the response measured.

**Figure 7:**
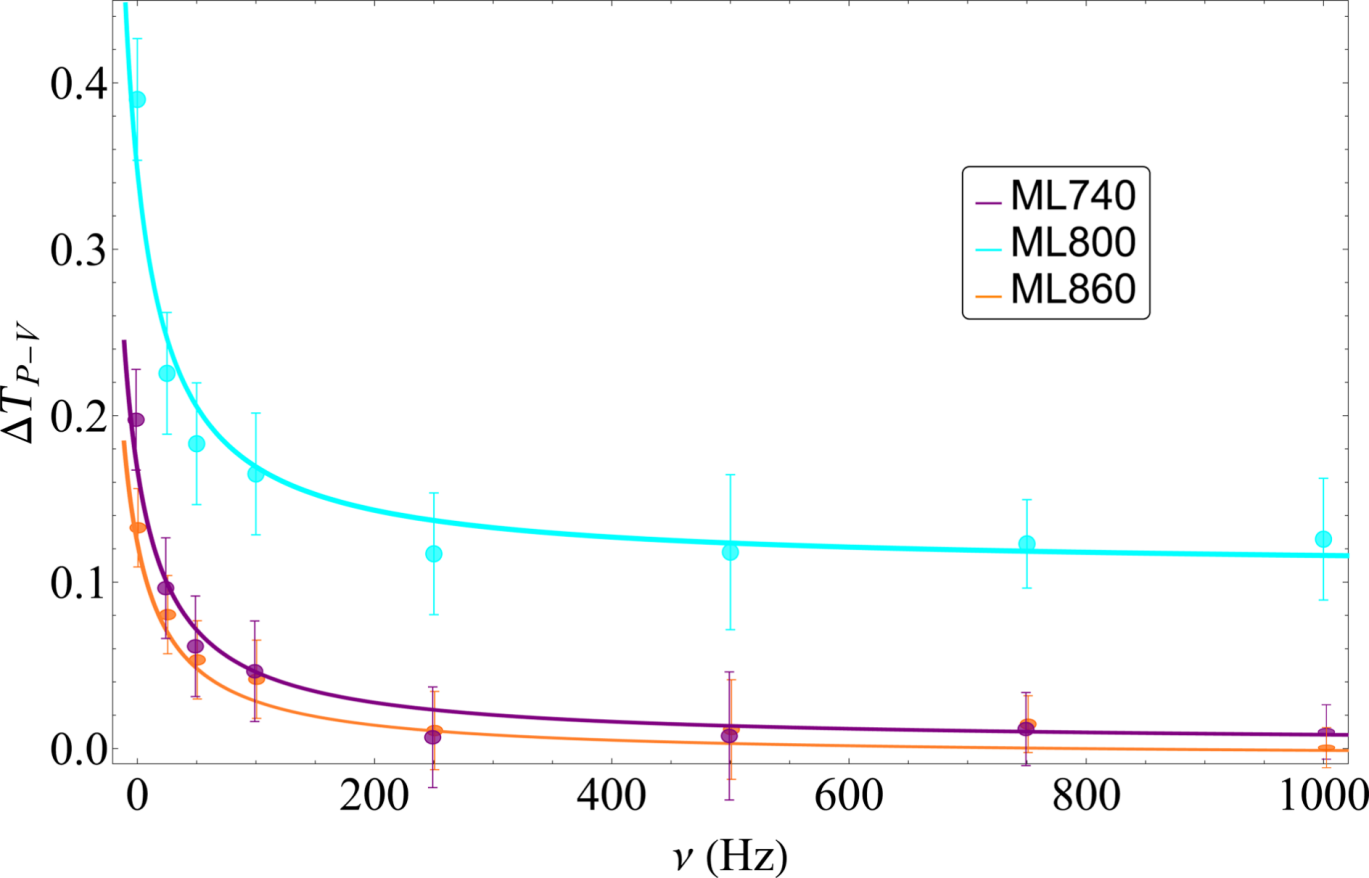
Peak-to-valley transmittance differences Δ*T*_P−V_ as a function of the chopper frequency for the samples ML740 (purple), ML800 (cyan), and ML860 (orange). The solid line is the fit using the equation provided in [32], following the procedure explained in [24]. When the chopper is turned off, we have the highest accumulated thermal effect; as the frequency rises, we see that the thermal effect gradually decreases until Δ*T*_P−V_ is constant. Hence, it can be assumed that the thermal effects were successfully suppressed.

Using the modification of the z-scan technique proposed in [30], we studied the samples ML740, ML800, and ML860. Figure 7 shows the Δ*T*_P−V_ values extracted from the CA data at *I*_0_ = 1.09 GW·cm^−2^ as a function of the chopper frequency. We obtained the fit using the equation provided in [32], following the procedure explained in [24]. We observe a maximum Δ*T*_P−V_ value for ν_chopper_ = 0, that is, when the whole pulse train is applied to the sample and, hence, the thermal load is maximal. When the chopper frequency is increased, Δ*T*_P−V_ diminishes and quickly reaches a stable value for chopper frequencies above 300 Hz. Therefore, for these higher ν_chopper_ values, we only have the electronic contribution. From this, we obtain the nonlinear refractive index of the electronic contribution 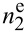; from the frequency ν_chopper_ ≈ 0, we obtain the value for both electronic and thermal contribution 
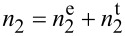
.

From Figure 7, we were able to differentiate the electronic and thermal contributions to the nonlinear response. In Table 3, we present the values with the corresponding nonlinear refractive indexes. We see that both contributions are positive, which is consistent with what we get without the chopper. We observe that, as expected, we have a thermal contribution to the nonlinear response. Regardless of this, the measured electronic contribution is significant. Compared to the nonlinear refractive index obtained from the normal z-scan technique, we do not observe any inconsistency with the values and order magnitude of the newly obtained nonlinear refractive indexes. We notice that the thermal contribution is around 70% of the nonlinear response for all three cases. This could mean that the pulse-to-pulse temperature accumulation occurs mainly in the gold layers, which have a fixed thickness in all three samples. We can also see that the electronic contribution to the nonlinear response follows the same trend as the nonlinear response measured with the full train of pulses; we obtain an enhancement in the ML800 sample of up to two orders of magnitude with respect to the other samples. Given the thermal contribution and the high repetition rate of the system, the material may be damaged or its properties may be changed due to heat accumulation. However, in repeated z-scan studies with the used peak irradiances, we did not see any damage, and repeating the experiment at the highest value gave the same signal each time.

**Table 3:** Nonlinear properties (electronic and thermal contributions to the nonlinear refractive index) of the multilayered stacks with *d*_m_ = 10 nm.

Sample	*n*_2_ (×10^−2^ cm^2^·GW^−1^)	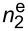 (×10^−2^ cm^2^·GW^−1^)	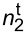 (×10^−2^ cm^2^·GW^−1^)	*t*_c_ (s)	*D* (×10^−6^ m^2^·s^−1^)

ML740	0.53 ± 0.03	0.37 ± 0.04	0.18 ± 0.01	0.011 ± 0.003	2.8 ± 0.3
ML800	10.3 ± 0.4	6.7 ± 0.5	2.5 ± 0.3	0.013 ± 0.001	2.4 ± 0.2
ML860	0.064 ± 0.006	0.045 ± 0.005	0.019 ± 0.003	0.014 ± 0.002	2.2 ± 0.3

In Table 3, we show the obtained characteristic thermal time *t*_c_(*z*) = ω^2^(*z*)/(4*D*), where ω(*z*) is the beam’s waist and *D* is the thermal diffusivity. From this fit, we obtained times that are several orders of magnitude longer than the repetition rate of the pulses, which is consistent with the assumption of accumulated pulse-to-pulse thermal effects. From the characteristic thermal time *t*_c_, we are able to obtain the thermal diffusivity *D* for these structures, which is closer to the titanium dioxide diffusivity *D* = 1.35 × 10^−6^ m^2^·s^−1^ [33] than to that of gold *D* = 1.17 × 10^−4^ m^2^·s^−1^ [34]. This can be because the gold/titanium dioxide ratio is 1:6, resulting in a thermal effect dominated by the thermal properties of titanium dioxide.

## Conclusion

We were able to design and fabricate a multilayered system that presents hyperbolic dispersion. Ellipsometric data indicate that the ENZ points were slightly shifted towards longer wavelengths from those that they were designed for, but they remained near the desired region. Also, from the ellipsometric results, we can imply that, even though the gold layer was very thin, we were able to deposit a uniform layer instead of “island-like” layers that form for smaller thicknesses [28].

We studied both the absorptive and the refractive contribution to the nonlinear response of the fabricated samples, using the closed aperture z-scan technique with 100 fs pulses at 800 nm. The results show a fairly large enhancement of the nonlinearity of the sample with an ENZ point at 800 nm with a nonlinear refractive index *n*_2_ = 0.103 cm^2^·GW^−1^. This value is orders of magnitude larger than those of the other samples, meaning that we obtained an enhancement of the nonlinear response at the desired wavelength. The thermal and electronic contributions to the response were resolved using a modified z-scan technique to yield 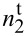 = 0.067 cm^2^·GW^−1^ and 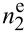 = 0.024 cm^2^·GW^−1^. Even though the thermal contribution dominates *n*_2_, 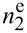 is comparable to those of other ENZ materials reported, such as Ru/TiO_2_ multilayers with *n*_2_ = 0.084 cm^2^·GW^−1^ [22], ITO with *n*_2_ = 0.11 cm^2^·GW^−1^ [35], AZO with *n*_2_ = 5.17 × 10^−3^ cm^2^·GW^−1^ [36], and a Au nanorod metamaterial with *n*_2_ = −0.024 cm^2^·GW^−1^ [37].

## Data Availability

Additional research data generated and analyzed during this study is not shared.
